# Synthesis and biological evaluation of heterocyclic 1,2,4-triazole scaffolds as promising pharmacological agents

**DOI:** 10.1186/s13065-020-00717-y

**Published:** 2021-01-21

**Authors:** Mukesh Kumari, Sumit Tahlan, Balasubramanian Narasimhan, Kalavathy Ramasamy, Siong Meng Lim, Syed Adnan Ali Shah, Vasudevan Mani, Saloni Kakkar

**Affiliations:** 1 Faculty of Pharmaceutical Sciences, Maharshi Dayanand University, Rohtak, 124001 India; 2 Faculty of Pharmacy, Universiti Teknologi MARA (UiTM), 42300 Bandar Puncak Alam, Selangor Darul Ehsan Malaysia; 3 Collaborative Drug Discovery Research (CDDR) Group, Pharmaceutical Life Sciences Community of Research, Universiti Teknologi MARA (UiTM), 40450 Shah Alam, Selangor Darul Ehsan Malaysia; 4 Atta-Ur-Rahman Institute for Natural Products Discovery (AuRIns), Universiti Teknologi MARA (UiTM), Puncak Alam Campus, 42300 Bandar Puncak Alam, Selangor Darul Ehsan Malaysia; 5 Department of Pharmacology and Toxicology, College of Pharmacy, Qassim University, Buraidah, 51452 Kingdom of Saudi Arabia

**Keywords:** 1,2,4-Triazole, Antimicrobial, Antioxidant, Anti-*urease*, Anticancer, SAR

## Abstract

**Background:**

Triazole is an important heterocyclic moiety that occupies a unique position in heterocyclic chemistry, due to its large number of biological activities. It exists in two isomeric forms i.e. 1,2,4-triazole and 1,2,3-triazole and is used as core molecule for the design and synthesis of many medicinal compounds. 1,2,4-Triazole possess broad spectrum of therapeutically interesting drug candidates such as analgesic, antiseptic, antimicrobial, antioxidant, anti-*urease*, anti-inflammatory, diuretics, anticancer, anticonvulsant, antidiabetic and antimigraine agents.

**Methods:**

The structures of all synthesized compounds were characterized by physicochemical properties and spectral means (IR and NMR). The synthesized compounds were evaluated for their in vitro antimicrobial activity against Gram-positive (*B. subtilis*), Gram-negative (*P. aeruginosa* and *E. coli*) bacterial and fungal (*C. albicans* and *A. niger*) strains by tube dilution method using ciprofloxacin, amoxicillin and fluconazole as standards. In-vitro antioxidant and anti-*urease* screening was done by DPPH assay and indophenol method, respectively. The in-vitro anticancer evaluation was carried out against MCF-7 and HCT116 cancer cell lines using 5-FU as standards.

**Results, discussion and conclusion:**

The biological screening results reveal that the compounds **T**_**5**_ (MIC_*BS*, *EC*_ = 24.7 µM, MIC_*PA*_, _*CA*_ = 12.3 µM) and **T**_**17**_ (MIC_*AN*_ = 27.1 µM) exhibited potent antimicrobial activity as comparable to standards ciprofloxacin, amoxicillin (MIC_Cipro_ = 18.1 µM, MIC_Amo_ = 17.1 µM) and fluconazole (MIC_Flu_ = 20.4 µM), respectively. The antioxidant evaluation showed that compounds **T**_**2**_ (IC_50_ = 34.83 µg/ml) and **T**_**3**_ (IC_50_ = 34.38 µg/ml) showed significant antioxidant activity and comparable to ascorbic acid (IC_50_ = 35.44 µg/ml). Compounds **T**_**3**_ (IC_50_ = 54.01 µg/ml) was the most potent *urease* inhibitor amongst the synthesized compounds and compared to standard thiourea (IC_50_ = 54.25 µg/ml). The most potent anticancer activity was shown by compounds **T**_**2**_ (IC_50_ = 3.84 μM) and **T**_**7**_ (IC_50_ = 3.25 μM) against HCT116 cell lines as compared to standard 5-FU (IC_50_ = 25.36 μM).

## Introduction

**Fig. 1:**
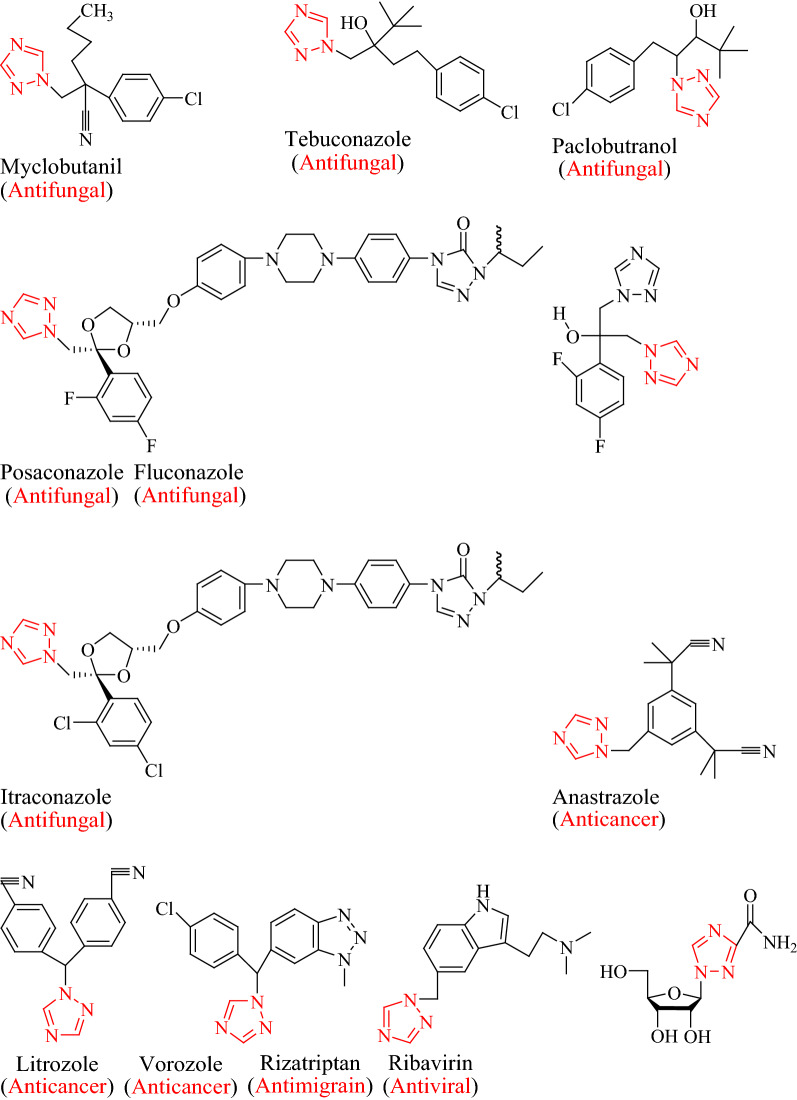
Marketed preparations containing 1,2,4-triazole as core moiety

Triazole is an N-bridged aromatic heterocyclic compound that received a considerable attention in recent years due to their biological activities [1]. The name “triazole” was first use by Bladin in 1855 for describing the carbon–nitrogen ring system C_2_H_3_N_3_ [2]. It is a white to pale yellow crystalline solid with a weak, characteristic odour, soluble in water and alcohol, melts at 120 °C and boils at 260 °C [3]. Triazole exists in two isomeric forms such as 1,2,4-triazole and 1,2,3-triazole [4]. The SAR studies of triazole derivative reveals that substitution on positions 3, 4 and 5 of triazole ring can be varied but the greatest changed in physicochemical properties and biological profile is exerted by the groups attached to the nitrogen atom at the 4th position [3]. It favours the hydrogen bonding and is also stable for metabolic degradation, which could be favorable in increasing solubility as well as in binding bimolecular targets [5]. Novel triazole drugs discovered and developed by applying bioisosteric replacement technique with extending biological activities also captured a special attention in medicinal chemistry [6]. Numerous medicines containing triazole moiety available in market (Fig. 1) are: *Antifungal* [7–10]—myclobutanil, tebuconazole, posaconazole, itraconazole fluconazole, paclobutrazole *Anticancer* [9, 11]—anastrazole, litrozole, vorozole, *Antimigrain* [9, 12]—rizatriptan and *Antiviral* [9, 13]—ribavirin.

At present time, our medical field is suffering from the problem of antimicrobial resistance towards many microbial strains. Hence as prioritized by various health organizations, there is a need for the discovery or development of novel antimicrobial compounds possessing a broad spectrum activity exhibiting high effectiveness against those highly resistant Gram positive, Gram negative bacterial and fungal strains [14].

Human cells face threats everyday because the attack of various viruses, infections and free radicals damage the body cells and DNA. Scientists observed that the free radicals contribute to the ageing process and also contribute in diseases, like cancer, diabetes and heart disease. Antioxidants are the chemicals that stop or limit the damage caused by the free radicals and also boost our immunity [15].

*Ureases* relate to the class of Urea amidohydrolases enzymes containing two nickel(II) atoms. *Ureases* are mainly obtained from plants, algae, fungi and bacteria. Bacterial *ureases* are responsible for causing many diseases like pyelonephritis, hepatic coma, peptic ulceration, urinary stones and stomach cancer. Rationally, A category of anti*urease* or *urease* inhibitory drugs was developed for curing the *urease* caused disease by inhibiting *urease* enzymes. The two nickel(II) atoms present in active site of *Ureases* accelerate the hydrolysis of urea into ammonia and carbon dioxide gas. Both CO_2_ and NH_3_ are important virulence factor for the pathogenesis of many above given clinical conditions. Anti-*urease* compounds inhibit the hydrolysis of urea by antagonising *urease* enzyme [16]. This article also focuses on some new 1,2,4-triazole derivatives exhibiting anti-*urease* activity.

Colorectal cancer is the third most lethal cancer worldwide in both males and females with drug resistance and metastasis being the major challenge to effective treatments. Maximum deaths due to colon cancer are related with metastatic ailment. The growth of colorectal cancer is promoted by epigenetic factors, such as abnormal DNA methylation. Targeted therapy is a kind of chemotherapy that specifically targets the proteins that resist the development of some cancers [17].

Palmitic acid (common name) is categorized as saturated fatty acid with chemical formula CH_3_(CH_2_)_14_COOH (IUPAC name: hexadecanoic acid). The main sources of palmitic acid are palm oil, olive oil, meats, cheese, cocoa butter, breast milk and dairy products [18]. Napalm, is a derivative of palmitic acid, synthesized by the combination of aluminium salts of palmitic acid and naphthenic acid and it was used as fuel during World War II [19].

**Fig. 2:**
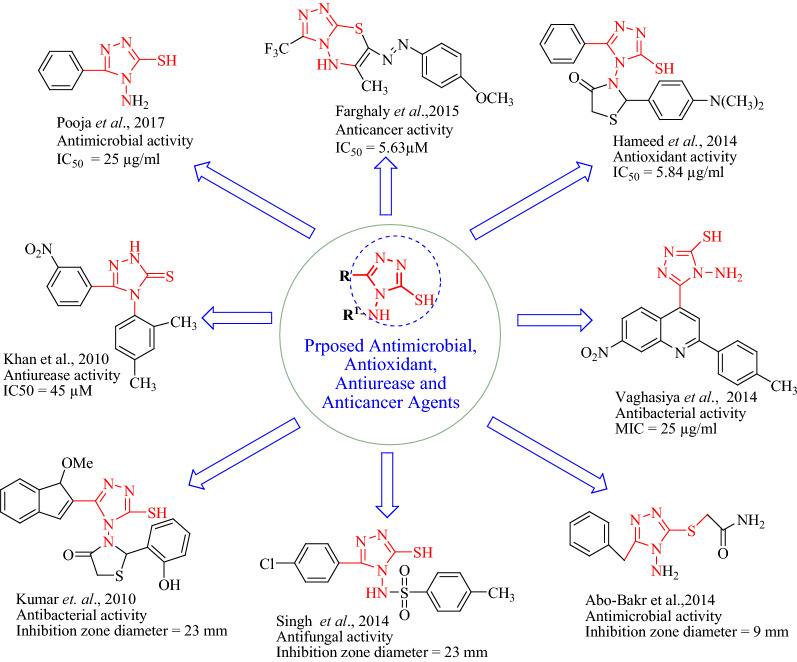
Design of 1,2,4-triazole analogues for antimicrobial, antioxidant, anti*urease* and anticancer activities based on biological profile

1,2,4-Triazole attracts the attention of researchers due to its broad spectrum of biological activities (Fig. 2) such as antimigrain [9, 12], antioxidant [15], anti-*urease* [16], antimicrobial [20, 21], anti-inflammatory [21, 22], anticonvulsant [23], anticancer [11, 24], antiviral [25] and antiparasytic [25].

## Results and discussion

### Chemistry

**Scheme 1.**
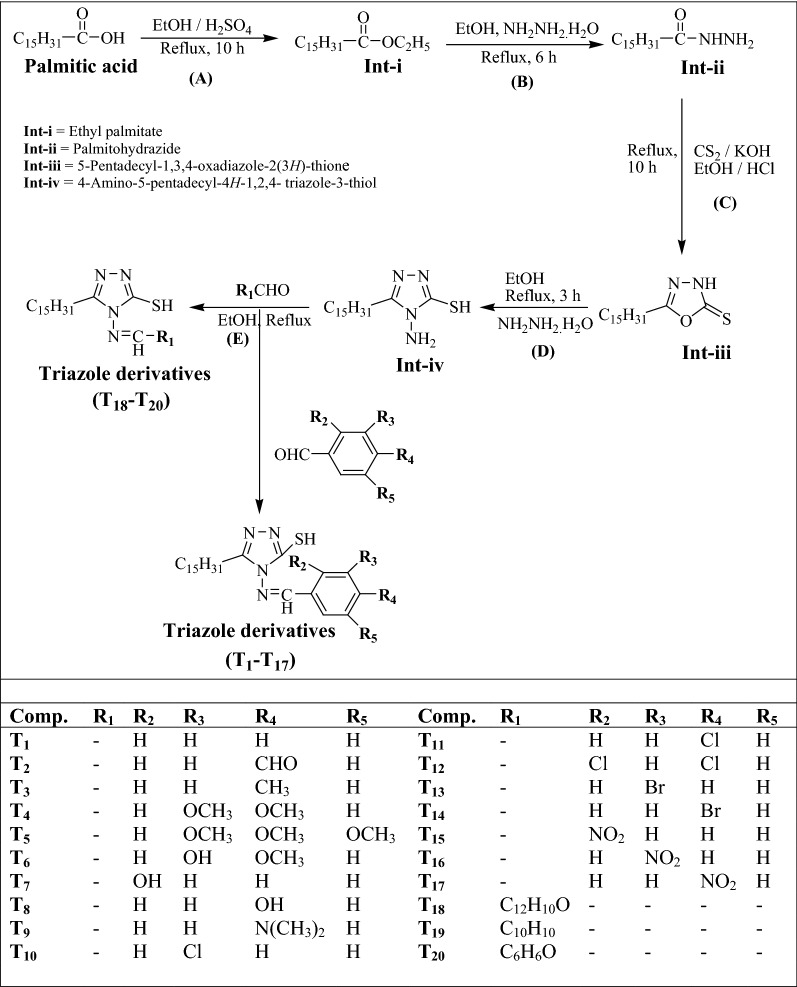
Synthesis of 1,2,4-triazole derivatives

The multistep synthetic process of 1,2,4-triazole derivatives (**T**_**1**_–**T**_**20**_) was depicted in Scheme 1. Initially, ethylpalmitate (**Int-i**) was synthesized by the reaction of palmitic acid, ethanol and sulphuric acid. Palmitohydrazide (**Int-ii**) was synthesized from ethanolic solution of ethylpalmitate (**Int-i**) followed by addition of hydrazine hydrate. 5-Pentadecyl-1,3,4-oxadiazole-2(3*H*)-thione (**Int-iii**) was synthesized using **Int-ii** in alc. potassium hydroxide solution followed by the addition of carbon disulfide and then followed by addition of hydrazine hydrate to **Int-iii** yielded 4-amino-5-pentadecyl-4*H*-1,2,4-triazole-3-thiol (**Int-iv**). Finally, the **Int-iv** on reaction with different substituted aromatic aldehydes in ethanol yielded the title compounds (**T**_**1**_–**T**_**20**_). The physicochemical properties of the synthesized compounds are depicted in Table 1. The synthesized derivatives of 1,2,4-triazole were confirmed by Infrared (IR) and Nuclear Magnetic Resonance (^1^H/^13^CNMR). The spectro-analytical data has been depicted in Table 2. The presence of aliphatic –CH– stretch in all compounds was confirmed at 2990–2879 cm^−1^. The intermediates (**Int-ii**, **iii** and **iv**) exhibited the –NH stretch in range of 3424–3319 cm^−1^. The presence of –CONH– group in **Int**-**ii** was indicated by appearance of –CONH– stretch at 1630 cm^−1^. The peak range 1677–1589 cm^−1^ in **Int-iii**, **iv** and compounds **T**_**1**_–**T**_**20**_ indicated the presence of –C=N stretch. The presence of –SH stretching vibrations in **Int-iv** and compounds **T**_**1**_–**T**_**20**_ were indicated in a scale of 2593–2505 cm^−1^. The compounds **T**_**4**_, **T**_**5**_ and **T**_**6**_ showed the –OCH_3_ stretching vibrations in the range of 2860–2848 cm^−1^. The presence of phenolic group in compounds **T**_**6**_, **T**_**7**_, **T**_**8**_ and **T**_**18**_ was indicated by peaks in the range of 3483–3400 cm^−1^. The peak range 701–699 cm^−1^ of compounds **T**_**13**_ and **T**_**14**_ was indicated the presence of Ar–Br group. The compounds **T**_**15**_, **T**_**16**_ and **T**_**17**_ showed the Ar–NO_2_ stretching vibrations in the range of 1545–1424 cm^−1^. The presence of Ar–Cl group in compounds **T**_**10**_, **T**_**11**_ and **T**_**12**_ was confirmed by the appearance of peaks in the range of 767–750 cm^−1^. The presence of tertiary amine in compound **T**_**9**_ was confirmed by the appearance of peak at 3431 cm^−1^. The presence of aromatic ring in compounds **T**_**1**_–**T**_**20**_ was indicated by the appearance of peak in the range of 1796–1719 cm^−1^. DMSO was used as solvent for the analysis of compounds by ^1^HNMR spectra. The presence of singlet signal at 1.22–2.47 δ ppm and 0.82–0.84 δ ppm indicated the presence of protons of –CH_2_ and –CH_3_ groups in **Int-ii**, **iii** and **iv**, respectively. Singlet at 2.25 δ ppm and 8.87 δ ppm showed the presence of protons of NH_2_ and NH groups in **Int**-**ii**, **iii** and **iv**, respectively. The presence of proton of SH group was indicated by appearance of singlet at 3.30 in **Int**-**iv**. The findings of elemental analysis of synthesized derivatives were recorded within theoretical results of ± 0.4%. Mass spectra of the synthesized derivatives reflected the characteristic molecular ion peaks.

**Table 1:** Physicochemical characterization of synthesized derivatives (**T**_**1**_–**T**_**20**_**)**

Comp	Mol. formula	Mol. wt	Colour	M.p. (°C)	Rf value	% Yield
**Int-ii**	C_18_H_36_O	284.84	White	121–124	0.37^a^	71.0
**Int-iii**	C_17_H_31_N_2_OS	312.51	Yellow	154–157	0.42^a^	52.1
**Int-iv**	C_17_H_34_N_4_S	326.54	Pinkish white	178–181	0.41^b^	46.0
**T**_**1**_	C_24_H_38_N_4_S	414.65	Creamish white	184–187	0.40^b^	78.0
**T**_**2**_	C_25_H_38_N_4_OS	442.66	Light brown	182–185	0.33^b^	82.0
**T**_**3**_	C_25_H_40_N_4_S	428.68	Yellowish white	190–193	0.52^b^	74.0
**T**_**4**_	C_26_H_43_N_4_O_2_S	474.70	White	188–191	0.60^b^	85.0
**T**_**5**_	C_27_H_44_N_4_O_3_S	504.73	Yellow	187–190	0.38^b^	79.5
**T**_**6**_	C_25_H_40_N_4_O_2_S	460.68	Creamish white	189–192	0.49^b^	84.6
**T**_**7**_	C_24_H_38_N_4_OS	430.65	Lemon yellow	181–184	0.32^b^	78.0
**T**_**8**_	C_24_H_38_N_4_OS	430.65	Greenish white	192–195	0.73^b^	65.9
**T**_**9**_	C_26_H_43_N_5_S	457.72	Green	198–201	0.62^b^	75.0
**T**_**10**_	C_24_H_37_ClN_4_S	449.10	Pinkish white	205–208	0.59^b^	79.6
**T**_**11**_	C_24_H_37_ClN_4_S	449.10	White	202–205	0.65^b^	78.0
**T**_**12**_	C_24_H_36_Cl_2_N_4_S	483.54	White	211–214	0.34^b^	74.3
**T**_**13**_	C_24_H_37_BrN_4_S	493.55	White	197–200	0.39^b^	89.2
**T**_**14**_	C_24_H_37_BrN_4_S	493.55	Brownish yellow	193–196	0.71^b^	85.8
**T**_**15**_	C_24_H_37_N_5_O_2_S	459.65	Mustard yellow	195–198	0.68^b^	78.9
**T**_**16**_	C_24_H_37_N_5_O_2_S	459.65	Dark yellow	199–202	0.45^b^	90.0
**T**_**17**_	C_24_H_37_N_5_O_2_S	459.65	Brown	206–209	0.50^b^	85.8
**T**_**18**_	C_28_H_40_N_4_OS	480.71	Red	186–189	0.67^b^	88.8
**T**_**19**_	C_26_H_40_N_4_OS	440.69	Creamish white	179–182	0.24^b^	87.9
**T**_**20**_	C_22_H_36_N_4_OS	404.61	White	196–199	0.43^b^	87.0

Mobile phase: ^a^[chloroform:toluene, 7:3], ^b^[ethylacetate:*n*-hexane, 2:3]

**Table 2:** Spectral characterization of synthesized derivatives (**T**_**1**_–**T**_**20**_)

Comp	IR (KBr, cm^−1^)	^1^H NMR (400 MHz, DMSO-*d*_*6*_)	^13^C NMR (400 MHz, DMSO-*d*_*6*_)	C, H, N analyses calculated (found); MS, ES + (ToF): *m/z*—[M^+^ + 1]
**Int-ii**	3319–3179 (N–H str.), 2920 (C–H str. aliphatic), 1739 (C=O str.)	1.22–2.24 (m, 28H, CH_2_), 0.83 (s, 3H, CH_3_), 2.5 (s, 2H, NH_2_), 8.873 (s, 1H, NH)	14.0, 19.4, 29.4, 39.1, 128.9, 149.9, 168.6, 189.4	C, 71.06; H, 12.67; N, 10.36; (C, 71.01; H, 12.57; N, 10.26); 271
**Int-iii**	3424 (N–H str.), 2921 (C–H str. aliphatic), 1372 (C=S str.), 1589 (C=N str.), 1115 (C–O str.)	1.22–2.47 (m, 28H, CH_2_), 0.84 (s, 3H, CH_3_)	15.7, 18.7, 29.8, 37.1, 124.1, 149.9, 160.6, 182.4	C, 65.34; H, 10.32; N, 8.96; (C, 65.31; H, 10.29; N, 8.91); 313
**Int-iv**	3319 (N–H str.), 2919 (C–H str., aliphatic), 1631 (C=N str.), 2572 (S–H str.)	1.22–2.12 (m, 28H, CH_2_), 0.82 (s, 3H, CH_3_), 2.51 (s, 2H, NH_2_), 8.87 (s, 1H, NH)	17.7, 18.4, 29.8, 39.6, 128.1, 124.98, 149.9, 168.6, 188.4	C, 62.53; H, 10.49; N, 17.16; (C, 62.50; H, 10.42; N, 17.12); 327
**T**_**1**_	2925 (C–H str., aliphatic), 1625 (C=N str.), 3100 (C=C str., aromatic), 1719 (C–H str., aromatic.), 2568 (S–H str.)	1.24–2.45 (m, 28H, CH_2_), 0.84 (s, 3H, CH_3_), 7.41–7.93 (m, 5H, Ar–H), 3.37 (s, H, SH)	15.0, 18.4, 29.1, 39.1, 124.1, 128.98, 148.9, 160.6, 182.4	C, 69.52; H, 9.24; N, 13.51; (C, 69.49; H, 9.22; N, 13.49); 415
**T**_**2**_	2990 (C–H str., aliphatic), 1646 (C=N str.), 1751 (C=O, str.), 1641 (C=C str., aromatic), 3073 (C–H str., aromatic), 2572 (S–H str.)	1.32–2.51 (m, 28H, CH_2_), 0.86 (s, 3H, CH_3_), 2.51 (s, 2H, CH_2_), 7.08–7.91 (m, 4H, Ar–H), 3.37 (s, H, SH), 10.02 (s, H, CHO)	13.8, 22.0, 28.5–28.9, 31.2, 129.8, 139.8, 140.9, 174.6, 192.4	C, 67.83; H, 8.65; N, 12.66; (C, 67.80; H, 8.61; N, 12.62); 443
**T**_**3**_	2922 (C–H str., aliphatic), 1649 (C=N str.), 1646 (C=C str., aromatic), 3098 (C–H str., aromatic), 1790 (ring, str.), 2560 (S–H str.)	1.29–2.51 (m, 24H, CH_2_), 0.87 (s, 3H, CH_3_), 7.30 (s, H, =CH), 7.51–7.62 (m, 4H, Ar–H), 3.37 (s, H, SH), 2.34 (s, 3H, Ar–CH_3_)	13.8, 22.0, 24.4, 28.5–28.8, 31.2, 128.6, 129.7, 142.3, 161.11	C, 70.05; H, 9.41; N, 13.07 (C, 70.00; H, 9.38; N, 13.02); 429
**T**_**4**_	2920 (C–H str., aliphatic), 1631 (C=N str.), 2856 (C–O–C str.), 1647 (C=C str., aromatic), 3105 (C–H str., aromatic), 2593 (S–H str)	1.26–2.51 (m, 28H, CH_2_), 0.86 (s, 3H, CH_3_), 7.90 (s, H, =CH), 7.01–7.61 (m, 3H, Ar–H), 3.11 (s, H, SH), 3.76 (s, 6H, Ar–OCH_3_)	13.8, 22.0, 28.3–28.9, 31.2, 55.1, 111.1, 149.1, 176.0	C, 65.78; H, 8.92; N, 11.80; (C, 65.72; H, 8.89; N, 11.78; O, 6.70); 475
**T**_**5**_	2923 (C–H str., aliphatic), 1639 (C=N str.), 2860 (C–O–C str.), 1450 (C=C str., aromatic), 3078 (C–H str., aromatic), 2572 (S–H str.)	1.23–2.47 (m, 28H, CH_2_), 0.85 (s, 3H, CH_3_), 7.21 (s, H, =CH), 7.10–7.27 (m, 2H, Ar–H), 3.52 (s, H, SH), 3.76 (s, 9H, Ar–OCH_3_)	14.0, 39.1, 105.6, 129.2, 140.2, 153.1, 161.1	C, 64.25; H, 8.79; N, 11.10; (C, 64.21; H, 8.75; N, 11.08); 505
**T**_**6**_	2988 (C–H str., aliphatic), 1677 (C=N str.), 1715 (ring, str.), 3400 (O–H str.), 2843 (C–O–C str.), 1636 (C=C str., aromatic), 3154 (C–H str., aromatic), 2555 (S–H str.)	1.21–2.46 (m, 28H, CH_2_), 0.96 (s, 3H, CH_3_), 8.0 (s, H,CH), 7.51–7.69 (m, 3H, Ar–H), 3.37 (s, H, SH), 6.71 (s, H, OH), 3.79 (s, H, Ar–OCH_3_)	13.8, 22.0, 28.5–28.9, 31.8, 55.5, 115.3, 120.7, 128.7, 142.7, 146.2, 168.2	C, 65.18; H, 8.75; N, 12.16; (C, 65.13; H, 8.78; N, 12.15); 461
**T**_**7**_	2923 (C–H str., aliphatic), 1617 (C=N str.), 3401(O–H, str.), 1632 (C=C str., aromatic), 3101 (C–H str., aromatic), 2695 (S–H str.)	1.27–2.52 (m, 28H, CH_2_), 0.85 (s, 3H, CH_3_), 8.35 (s, H, =CH), 7.31–7.79 m, 4H, Ar–H), 3.37 (s, H, SH), 4.05 (s, H, OH)	14.0, 15.0, 24.4, 29.0, 31.2, 33.9, 39.9, 59.9, 61.1, 116.6, 129.4, 157.06, 162.7	C, 66.94; H, 8.89; N, 13.01; (C, 66.90; H, 8.83; N, 12.9); 431
**T**_**8**_	2919 (C–H str., aliphatic), 1686 (C=N str.), 3483 (O–H, str.), 1665 (C=C str., aromatic), 3098 (C–H str., aromatic), 2572 (S–H str.)	1.28–2.53 (m, 28H, CH_2_), 0.88 (s, 3H, CH_3_), 8.10 (s, H, =CH), 6.43–7.49 (m, 4H, Ar–H), 3.37 (s, H, SH), 5.01 (s, H, OH)	14.3, 24.7, 29.0, 31.6, 34.1, 39.9, 46.2, 116.2, 129.1, 160, 175, 191.5	C, 66.94; H, 8.89; N, 13.01; (C, 66.90; H, 8.85; N, 12.89); 431
**T**_**9**_	2925 (C–H str., aliphatic), 1684 (C=N str.), 1645 (C=C str., aromatic), 3097 (C–H str., aromatic), 3431 (N–H amine, str.) 2572 (S–H str.)	1.21–2.51 (m, 28H, CH_2_), 0.85 (s, 3H, CH_3_), 7.11–7.48 (m, 4H, Ar–H), 8.12 (s, H, =CH), 3.01 (s, H, SH), 2.99 (s, 6H, N–(CH_3_)_2_)	13.8, 22.0, 24.4, 29.0, 31.2, 46.3, 57.6, 118.7, 124.9, 128.1, 130.1, 134.9, 168.1, 114.4	C, 68.23; H, 9.47; N, 15.30 (C, 68.20; H, 9.42; N, 15.27);458
**T**_**10**_	2916 (C–H str., aliphatic), 1650 (C=N str.), 1456 (C=C str., aromatic), 3065 (C–H str., aromatic), 750 (C–Cl str.), 2567 (S–H str.)	1.24–2.45 (m, 28H, CH_2_), 0.85 (s, 3H, CH_3_), 8.01 (s, H, =CH), 6.80–7.61 (m, 4H, Ar–H), 3.0 (s, H, SH)	14.2, 22.2, 24.5, 29.4, 31.0, 46.3, 57.6, 118.7, 128.1, 130.1, 134.9, 148, 168.1	C, 64.19; H, 8.30; N, 12.48; (C, 64.15; H, 8.28; N, 12.43); 449
**T**_**11**_	2916 (C–H str., aliphatic), 1650 (C=N str.), 1635 (C=C str., aromatic), 3105 (C–H str., aromatic), 767 (C–Cl str.), 2572 (S–H str.)	1.26–2.56 (s, 28H, CH_2_), 0.87 (s, 3H, CH_3_), 8.01 (s, H, =CH), 6.90–7.68 (m, 4H, Ar–H), 3.2 (s, H, SH)	14.1, 22.0, 24.2, 29.9, 31.9, 40.3, 55.6, 118.7, 128.1, 131.1, 137.9, 148.9, 168.8	C, 64.19; H, 8.30; N, 12.48; (C, 64.15; H, 8.27; N, 12.43); 449
**T**_**12**_	2905 (C–H str., aliphatic), 1678.98 (C=N str.), 1604 (C=C str., aromatic), 3087 (C–H str., aromatic), 767 (C–Cl str.), 2505 (S–H str.)	1.23–2.51 (m, 28H, CH_2_), 0.86 (s, 3H, CH_3_), 8.10 (s, H, =CH), 6.60–7.57 (m, 3H, Ar–H), 3.07 (s, H, SH)	13.9, 22.5, 24.4, 29.9, 31.5, 44.3, 57.6, 119.7, 127.1, 131.9, 139.9, 148.9, 168.9	C, 59.61; H, 7.50; N, 11.59; (C, 59.58; H, 7.48; N, 11.53); 483
**T**_**13**_	2919 (C–H str., aliphatic), 1648 (C=N str.), 1624 (C=C str., aromatic), 3109 (C–H str., aromatic), 699 (C–Br str.), 2505 (S–H str)]	1.29–0.55 (m, 28H, CH_2_), 0.87 (s, 3H, CH_3_), 8.12 (s, H, =CH), 6.98–7.65 (m, 4H, Ar–H), 3.02 (s, H, SH)	14.4, 23.2, 24.0, 29.9, 33.3, 48.3, 59.6, 118.7, 128.9, 130.9, 134.8, 148.7, 168.6	C, 58.41; H, 7.56; N, 11.35; (C, 58.41; H, 7.56; N, 11.35); 495
**T**_**14**_	2919 (C–H str., aliphatic), 1648 (C=N str.), 1498 (C=C str., aromatic), 3054 (C–H str., aromatic), 699 (C–Br str.), 2505 (S–H str)	1.29–2.59 (m, 28H, CH_2_), 0.88 (s, 3H, CH_3_), 8.98 (s, H, =CH), 6.50–7.68 (m, 4H, Ar–H), 3.09 (s, H, SH)	15.4, 23.8, 24.9, 29.9, 33.0, 47.3, 56.6, 116.7, 129.9, 131.2, 134.8, 148.8, 168.9	C, 58.41; H, 7.56; N, 11.35; (C, 58.39; H, 7.53; N, 11.30); 495
**T**_**15**_	2921 (C–H str., aliphatic), 1698 (C=N str.), 1445 (C=C str., aromatic), 3100 (C–H str., aromatic), 1545 (NO_2_ str.), 2529 (S–H str.)	1.29–2.67 (m, 28H, CH_2_), 0.87 (s, 3H, CH_3_), 8.02 (s, H, =CH), 7.51–7.64 (m, 4H, Ar–H), 3.07 (s, H, SH),	13.4, 23.7, 24.5, 29.9, 32.3, 48.6, 59.8, 118.9, 127.9, 131.9, 134.9, 148.7, 168.5	C, 62.71; H, 8.11; N, 15.24; (C, 62.69; H, 8.09; N, 15.20); 460
**T**_**16**_	2878 (C–H str., aliphatic), 1648 (C=N str.), 1646 (C=C str., aromatic), 3045 (C–H str., aromatic), 1424 (NO_2_ str.), 2572 (S–H str.)	1.29–2.51 (m, 28H, CH_2_), 0.87 (s, 3H, CH_3_), 8.01 (s, H, CH), 7.51–7.96 (m, 4H, Ar–H), 3.37 (s, H, SH), 2.34 (s, 3H, Ar–CH_3_)	13.4, 23.7, 24.5, 29.9, 32.3, 48.6, 59.8, 118.9, 127.9, 131.9, 134.9, 148.7	C, 62.71; H, 8.11; N, 15.24; (C, 62.68; H, 8.07; N, 15.19); 460
**T**_**17**_	2880 (C–H str., aliphatic), 1647 (C=N str.), 1498 (C=C str., aromatic), 3076 (C–H str., aromatic), 1520 (NO_2_ str.), 2572(S–H str.)	1.24–2.56 (m, 28H, CH_2_), 0.86 (s, 3H, CH_3_), 8.31 (s, H, =CH), 6.90–7.76 (m, 4H, Ar–H), 3.07 (s, H, SH)	14.4, 23.9, 24.7, 29.8, 31.3, 48.6, 57.8, 116.9, 127.9, 131.7, 135.9, 148.9, 168.3	C, 62.71; H, 8.11; N, 15.24; (C, 62.68; H, 8.10; N, 15.22); 460
**T**_**18**_	2880 (C–H str., aliphatic), 1650 (C=N str.), 1608 (C=C str., aromatic), 3109 (C–H str., aromatic), 3401 (O–H str.), 2572 (S–H str.)	1.29–2.98 (m, 28H, CH_2_), 0.87 (s, 3H, CH_3_), 8.01 (s, H, =CH), 6.88–8.08 (m, 6H, Ar–H), 3.07 (s, H, SH), 5.01 (s, H, Ar–OH)	13.4, 22.9, 24.8, 29.8, 31.3, 46.6, 54.8, 116.9, 126.9, 130.7, 135.9, 148.9, 169.3, 189.9	C, 69.96; H, 8.39; N, 11.66; (C, 69.91; H, 8.32; N, 11.64); 481
**T**_**19**_	2985 (C–H str., aliphatic), 1650 (C=N str.), 1490 (C=C str., aromatic), 3099 (C–H str., aromatic), 2572 (S–H str.)	1.29–2.98 (s, 28H, CH_2_), 0.87 (s, 3H, CH_3_), 5.60–7.06 (m, 3H, =CH),6.01–7.69 (m, 6H, Ar–H), 3.07 (s, H, SH)	14.4, 22.1, 29.5, 31.2, 54.8, 116.9, 126.9, 130.7, 135.9, 148.6, 164.3, 189.6	C, 70.86; H, 9.15; N, 12.71; (C, 70.82; H, 9.12; N, 12.62); 441
**T**_**20**_	2879 (C–H str., aliphatic), 1648 (C=N str.), 2879 (C–O–C, str.), 1678 (C=C str., aromatic), 3156 (C–H str., aromatic), 2572 (S–H str)	1.29–2.52 (m, 28H, CH_2_), 0.84 (s, 3H, CH_3_), 6.30–7.40 (m, 3H, Ar–H), 3.07 (s, H, SH),	14.7, 21.9, 25.8, 26.8, 31.1, 47.6, 54.5, 119.9, 130.1, 135.5, 158.9, 179.3, 188.9	C, 65.31; H, 8.97; N, 13.85; (C, 65.28; H, 8.91; N, 13.80); 405

### Structure activity relationship (SAR) studies

**Fig. 3:**
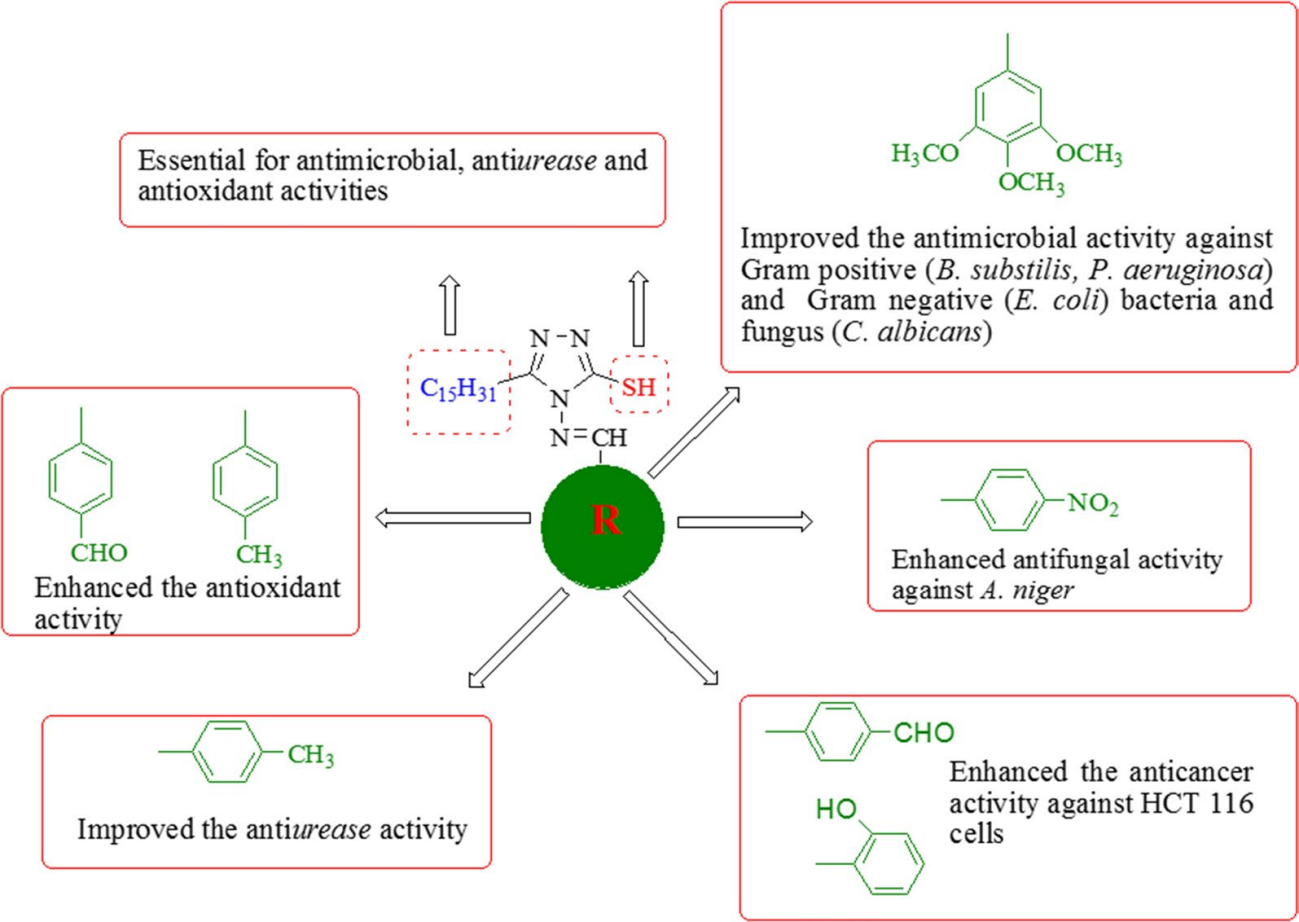
Structural requirements for antimicrobial, antioxidant and anti*urease* activity of 1,2,4-triazole derivatives

In the synthesized compounds, the substitution on *m-* and *p*-position of the aromatic ring with methoxy group improved the antimicrobial activity (compound **T**_**5**_, MIC_*BS*,_ _*EC*_ = 24.7 µM, MIC_*PA*,_ _*CA*_ = 12.3 µM) against Gram positive (*B. subtilis*, *P. aeruginosa*), Gram negative (*E. coli*) bacterial and fungal (*C. albicans*) strains, respectively. The *p*-substitution of nitro (compound **T**_**17**_, MIC_*AN*_ = 27.1 µM) group improved the antifungal activity against *A. niger*. The substituent methyl at *p*-position of ring (compound **T**_**3**_, IC_50_ = 54.01 µg/ml) enhanced the anti-*urease* activity. The antioxidant activity has been improved by *p*-substituents i.e. aldehyde (compound **T**_**2**_, IC_50_ = 34.83 µg/ml) and methyl groups (compound **T**_**3**_, IC_50_ = 34.38 µg/ml). The most potent anticancer activity showed by compounds **T**_**2**_ (IC_50_ = 3.84 μM) and **T**_**7**_ (IC_50_ = 3.25 μM) against HCT116 cell lines as compared to standard 5-FU (IC_50_ = 25.36 μM). From the analysis of antimicrobial activity, it may be concluded that the substitution of methoxy group increase the antibacterial activity whereas introduction of nitro as electron withdrawing groups at *p-*position may enhance the antifungal activity of synthesized compounds. The introduction of methyl substituent as electron donating groups at *p-*position of aromatic ring may increase the anti-*urease* as well as antioxidant activity. The substitution of of *p*-aldehyde and *o*-hydroxy group on the aromatic ring may enhance the anticancer activity against HCT116 cells (Fig. 3).

## Experimental

The initial material, reagents and solvents were purchased from Loba chemie. The glasswares were obtained from Borosil. The raw material was weighed on calibrated weighing balance. The synthetic scheme was drawn via ChemDraw 8.03. The confirmation of reaction at every step was done by TLC (thin layer chromatography). Melting point of the synthesized compounds was depicted by labtech melting point equipment. For spectral characterizations of the compounds, Bruker 12060280, Software: OPUS 7.2.139.1294 spectrometer using ATR for IR spectra (cm^−1^) and Bruker Avance III at 600 NMR and 150 MHz for ^1^H and ^13^CNMR (DMSO-*d6*, δ ppm) were used. The tested microbial strains like Gram positive, Gram negative bacteria and fungi were obtained from the Institute of Microbial Technology and Gene bank, Chandigarh for the in vitro antimicrobial activity. Waters Micromass Q-ToF Micro instrument was used for mass spectra. Elemental analysis was performed on Perkin-Elmer 2400 C, H and N analyzer and all synthesized compounds gave C, H and N analysis within ± 0.4% of the theoretical results.

### Procedure for synthesized 1,2,4-triazole derivatives (T_1_–T_20_)

#### Step A: synthesis of Int-i

A mixture of palmitic acid (2.6 g, 0.01 mol), absolute ethanol (50 ml) and few drops of conc. sulphuric acid (0.5 ml) was refluxed for 10 h in a round bottom flask and then cooled to 5 °C. The liquid product was separated from reaction mixture by using ether on the basis of density and then purified [26].

#### Step B: synthesis of Int-ii

To a solution of ethyl palmitate (**Int-i**, 2.8 g, 0.01 mol) in absolute ethanol (30 ml), hydrazine hydrate (0.64 g, 0.02 mol) was added and refluxed for 6 h and then left to cool. The solid product was collected by filtration and recrystallized from ethanol [26].

#### Step C: synthesis of Int-iii

Palmitohydrazide (**Int-ii**, 3.12 g, 0.01 mol) dissolved in the solution of potassium hydroxide (1.12 g, 0.02 mol) in ethanol (30 ml) and then (0.76 g, 0.01 mol) carbon disulfide was added slowly in the reaction mixture. The reaction mixture was refluxed for 10–12 hand then cooled at room temperature followed by addition of hydrochloric acid for neutralization of product. The precipitated solid was filtered, washed with ethanol, dried and recrystallized from ethanol [27].

#### Step D: synthesis of Int-iv

An ethanolic (30 ml) solution of 5-pentadecyl-1,3,4-oxadiazole-2(3*H*)-thione (**Int-iii**, 3.26 g, 0.01 mol) and hydrazine hydrate (0.38 g, 0.01 mol) was heated under reflux for 3 h and then solution was poured in ice. The resulting product was filtered, washed and recrystallized from ethanol [26, 27].

#### Step E: synthesis of 1,2,4-triazole derivatives (T_1_–T_20_)

The reaction mixture of 4-amino-5-pentadecyl-4*H*-1,2,4-triazole-3-thiol (**Int-iv**, 3.26 g, 0.01 mol) and different substituted aldehydes (0.01 mol) in ethanol followed by addition of few drops of sulphuric acid was refluxed for an appropriate time. The reaction was monitored by thin layer chromatography. After completion of reaction, the product was poured in ice and filtered, then wash and finally solid products were collected and recrystallized from ethanol [27].

### Biological studies

#### Antimicrobial evaluation

**Fig. 4:**
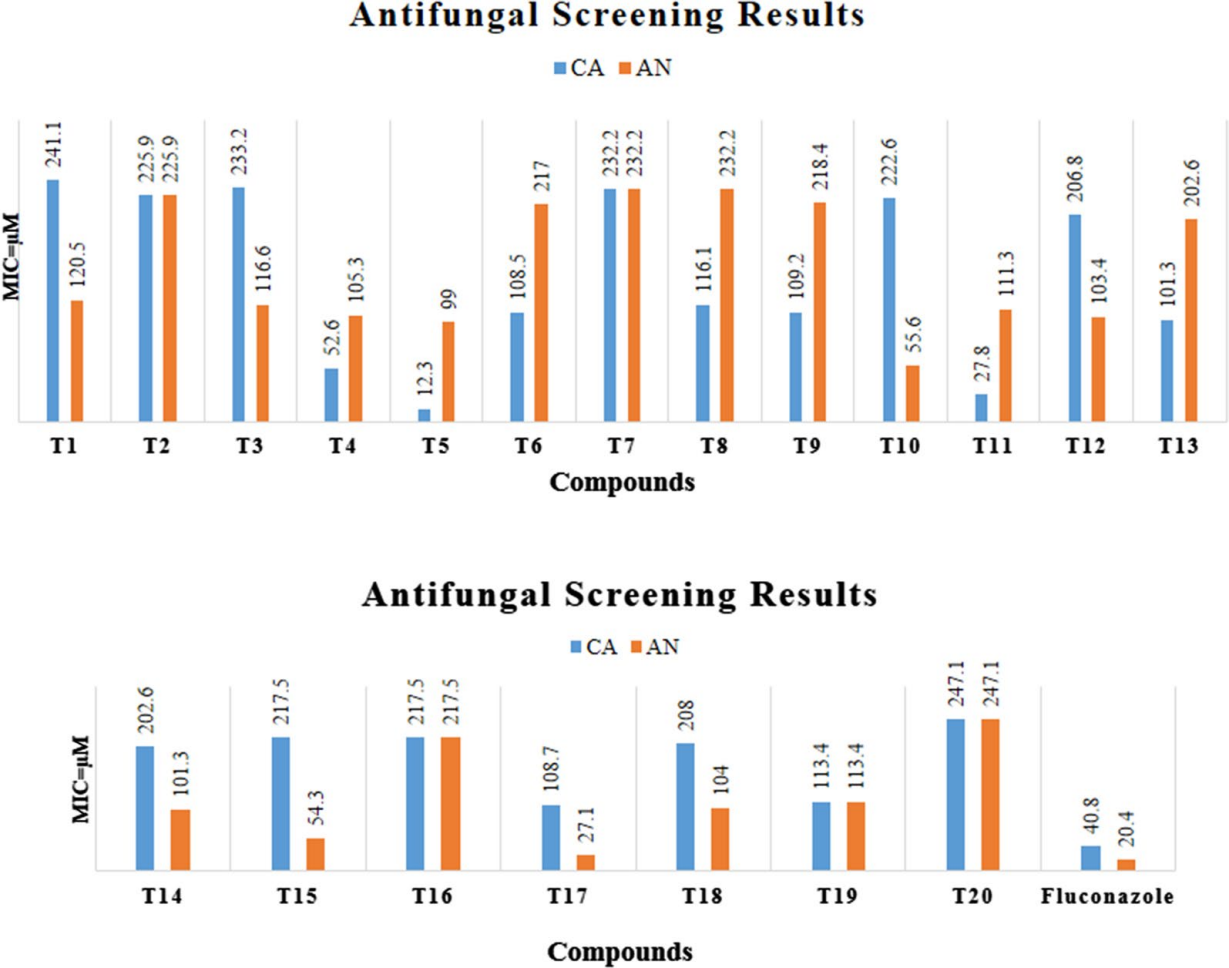
Antifungal screening results of synthesized derivatives (**T**_**1**_–**T**_**20**_)

**Fig. 5:**
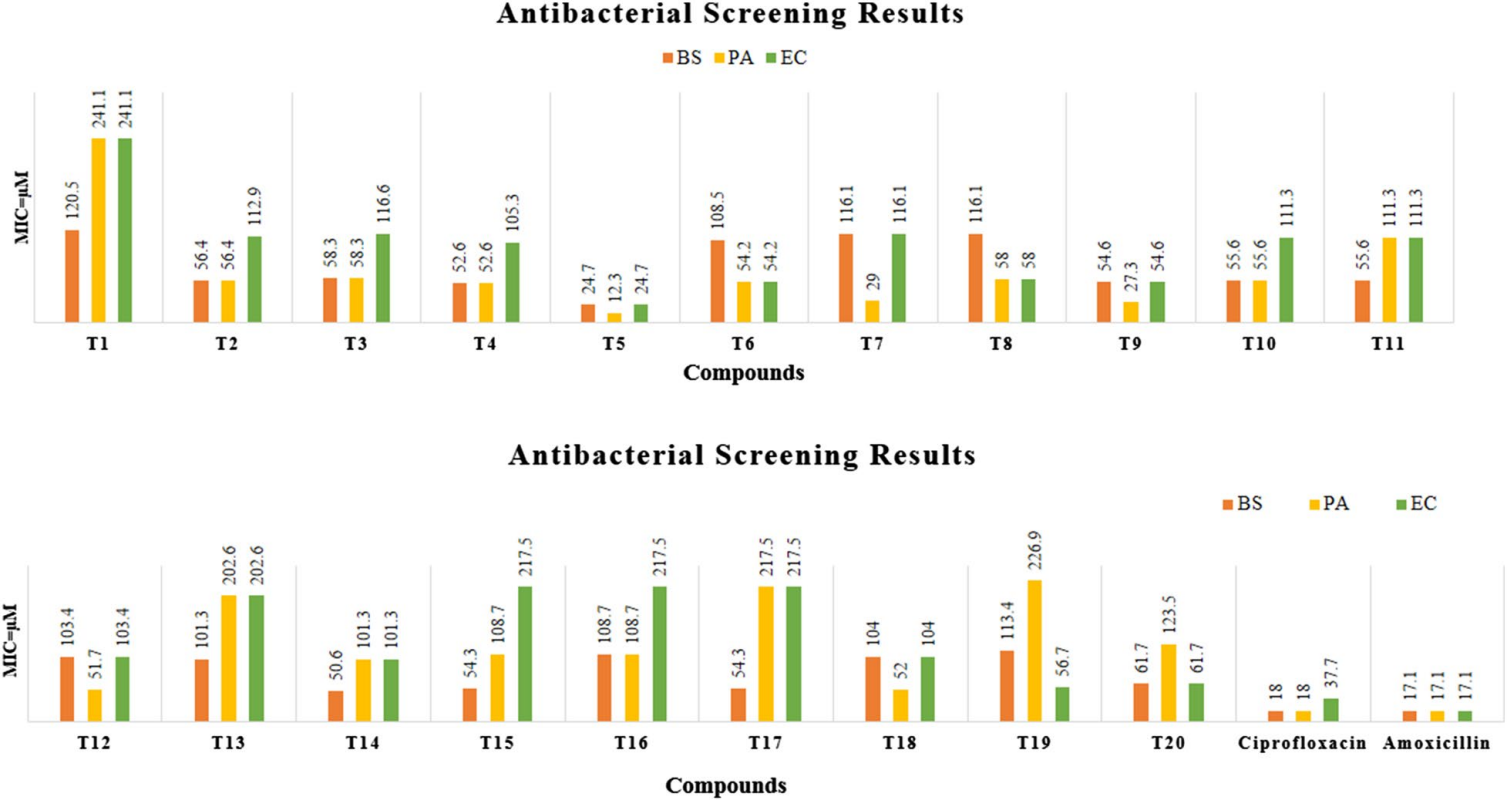
Antibacterial screening results of synthesized derivatives

The in vitro antimicrobial screening of the synthesized 1,2,4-triazole derivatives (**T**_**1**_–**T**_**20**_) in μM was determined against Gram-positive *Bacillus subtilis*, *Pseudomonas aeruginosa*, Gram-negative *Escherichia coli* bacterium and fungal strains *Candida albicans* and *Aspergillus niger* by tube dilution method using ciprofloxacin, amoxycillin (antibacterial) and fluconazole (antifungal) as reference drugs. DMSO was used to dissolve the reference and sample derivatives (**T**_**1**_–**T**_**20**_). Dilutions were prepared in nutrient broth (I.P.) for bacterial (incubated at 37 ± 1 °C for 24 h) and Sabouraud dextrose broth (I.P.) for fungal species (37 ± 1 °C for 48 h for *C. albicans*) and (25 ± 1 °C for 7 days for *A. niger*) (Table 3, Figs. 4 and 5) [17].

**Table 3:** Antimicrobial screening results of the synthesized 1,2,4-triazole derivatives (**T**_**1**_–**T**_**20**_**)**

Compound	Minimum inhibitory concentration (µM)				
	Bacterial strain			Fungal strain	
	Gram +ve	Gram −ve			
	*B. substilis*	*P. aeruginosa*	*E. coli*	*C. albican*	*A. niger*
**T**_**1**_	120.5	241.1	241.1	241.1	120.5
**T**_**2**_	56.4	56.4	112.9	225.9	225.9
**T**_**3**_	58.3	58.3	116.6	233.2	116.6
**T**_**4**_	52.6	52.6	105.3	52.6	105.3
**T**_**5**_	*24.7*	*12.3*	*24.7*	*12.3*	99.0
**T**_**6**_	108.5	54.2	54.2	108.5	217.0
**T**_**7**_	116.1	29.0	116.1	232.2	232.2
**T**_**8**_	116.1	58.0	58.0	116.1	232.2
**T**_**9**_	54.6	27.3	54.6	109.2	218.4
**T**_**10**_	55.6	55.6	111.3	222.6	55.6
**T**_**11**_	55.6	111.3	111.3	27.8	111.3
**T**_**12**_	103.4	51.7	103.4	206.8	103.4
**T**_**13**_	101.3	202.6	202.6	101.3	202.6
**T**_**14**_	50.6	101.3	101.3	202.6	101.3
**T**_**15**_	54.3	108.7	217.5	217.5	54.3
**T**_**16**_	108.7	108.7	217.5	217.5	217.5
**T**_**17**_	54.3	217.5	217.5	108.7	*27.1*
**T**_**18**_	104.0	52.0	104.0	208.0	104.0
**T**_**19**_	113.4	226.9	56.7	113.4	113.4
**T**_**20**_	61.7	123.5	61.7	247.1	247.1
**Fluconazole**	–	–	–	*40.8*	*20.4*
**Ciprofloxacin**	*18.0*	*18.0*	*37.7*	–	–
**Amoxicillin**	*17.1*	*17.1*	*17.1*	–	–

Italics signifies the most active compound in comparison to the standard compound

#### In vitro antioxidant evaluation

**Fig. 6:**
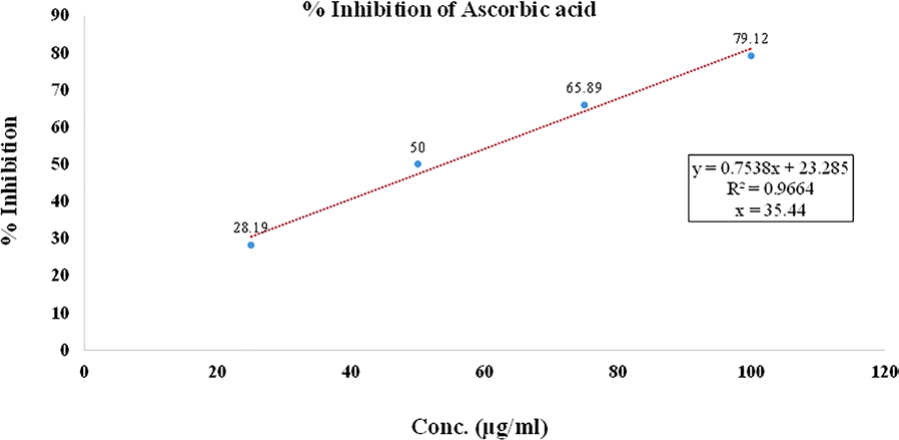
% inhibition of ascorbic acid

**Fig. 7:**
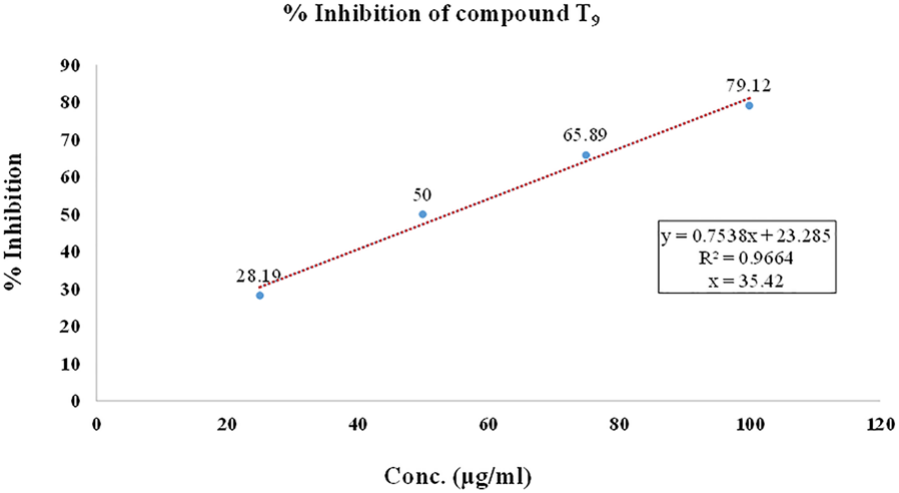
% inhibition of most active compound **T**_**9**_

**Fig. 8:**
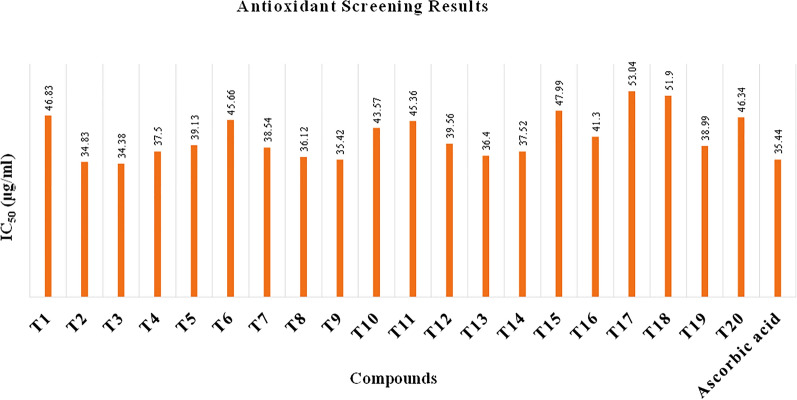
Antioxidant screening results of synthesized derivatives (**T**_**1**_–**T**_**20**_)

In the DPPH free radical scavenging activity, compounds (**T**_**1**_–**T**_**20**_) were evaluated for their free radical scavenging activity with ascorbic acid as standard compound. The IC_50_ was calculated for each compound as well as ascorbic acid as standard and summarized in Table 4 and shown in Figs. 6, 7, 8. The scavenging effect increased with the increasing concentrations of sample compounds. DPPH is relatively stable nitrogen centered free radical that easily accepts an electron or hydrogen radical to become a stable diamagnetic molecule. DPPH radicals react with suitable reducing agents as a result of which the electrons become paired off forming the corresponding hydrazine. The solution therefore loses colour stoichometrically depending on the number of electrons taken up. Fifty millilitres of various concentrations (25, 50, 70 and 100 µg/ml) of the compounds dissolved in methanol was added to 5 ml of a 0.004% methanolic solution of DPPH. The sample solutions were incubated for 30 min at room temperature in dark place and after then absorbance was recorded against the blank solution at 517 nm. The relative percent of DPPH scavenging activity was calculated according to the following equation:

$$ {\text{I}}\% = \frac{{{\text{A}}_{{{\text{control}}}} - {\text{A}}_{{{\text{sample}}}} }}{{{\text{A}}_{{{\text{control}}}} }} \times 100, $$

**Table 4:** Antioxidant screening results of the synthesized compounds (**T**_**1**_–**T**_**20**_**)**

Compounds	% inhibition				IC_50_ (µg/ml)
	25 (µg/ml)	50 (µg/ml)	75 (µg/ml)	100 (µg/ml)	
**T**_**1**_	25.45	49.65	69.67	94.78	46.83
**T**_**2**_	45.67	56.98	70.16	84.21	*34.83*
**T**_**3**_	43.56	60.12	75.67	88.98	*34.38*
**T**_**4**_	38.45	58.34	88.61	98.89	37.50
**T**_**5**_	42.34	55.67	68.98	78.12	39.13
**T**_**6**_	31.67	53.67	77.78	92.67	45.66
**T**_**7**_	46.75	52.56	60.56	70.41	38.54
**T**_**8**_	43.91	58.67	67.54	82.57	36.12
**T**_**9**_	40.56	60.67	83.61	92.16	35.42
**T**_**10**_	45.56	51.56	57.57	62.67	43.57
**T**_**11**_	35.78	52.89	70.89	89.9	45.36
**T**_**12**_	44.45	51.45	68.56	70.61	39.56
**T**_**13**_	43.46	57.67	73.76	89.56	36.40
**T**_**14**_	37.56	60.68	85.79	95.61	37.52
**T**_**15**_	38.56	50.64	62.16	80.56	47.99
**T**_**16**_	43.65	51.45	67.26	81.76	41.30
**T**_**17**_	48.75	62.57	78.16	84.61	24.90
**T**_**18**_	39.59	68.57	80.13	95.89	33.34
**T**_**19**_	38.45	58.34	80.61	96.89	38.99
**T**_**20**_	33.45	53.67	70.46	92.67	46.34
**Ascorbic acid**	38.67	63.68	84.78	94.45	*35.44*

Italics signifies the most active compound in comparison to the standard compound

where A_control_ is the absorbance of the control, A_sample_ is the absorbance of the test compound.

#### Urease inhibition evaluation

**Fig. 9:**
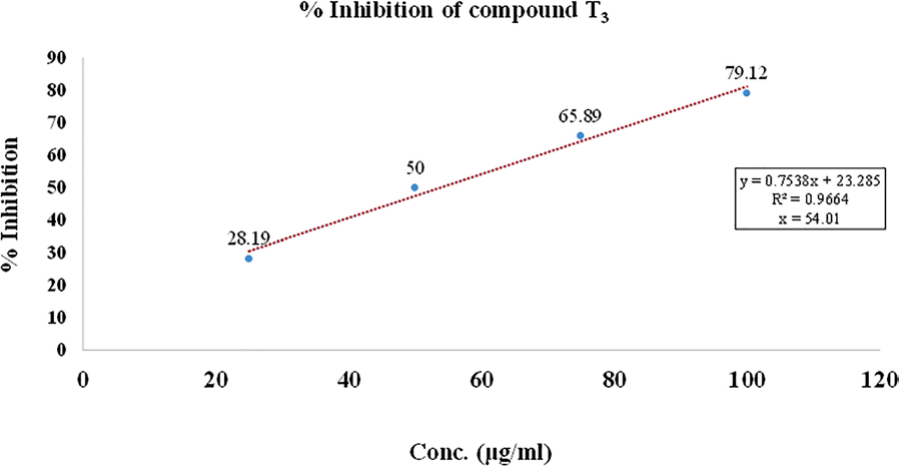
% inhibition of the most active compound **T**_**3**_

**Fig. 10:**
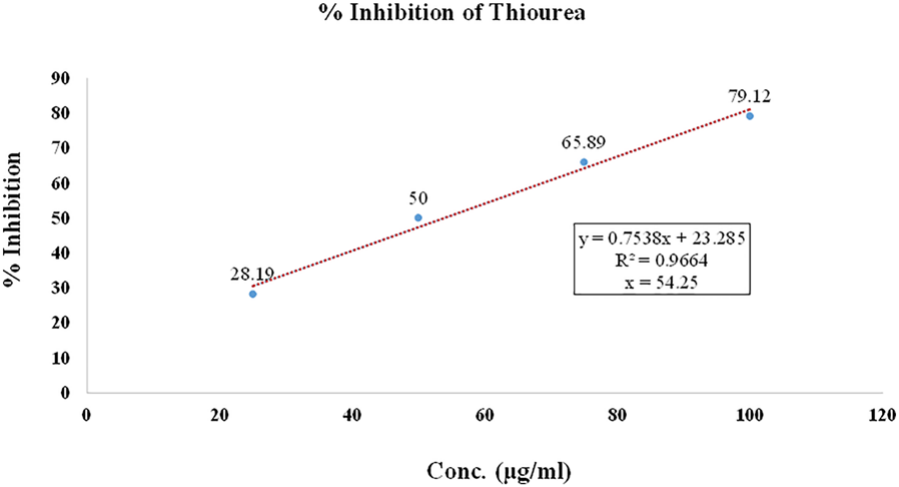
% inhibition of thiourea

**Fig. 11:**
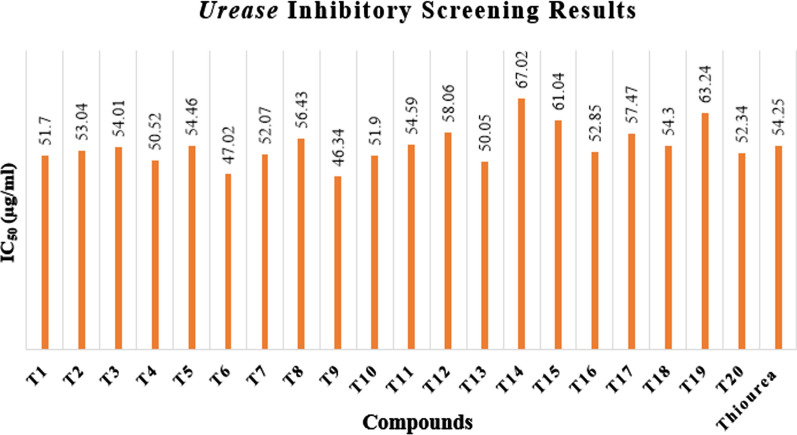
*Urease* inhibitory screening results of the synthesized derivatives (**T**_**1**_–**T**_**20**_)

*Urease* inhibitory potential for each synthesisized compound (**T**_**1**_–**T**_**20**_) was evaluated using Jack Bean *Urease* by Indophenol method (Table 5, Figs. 9, 10, 11). 250 µl of jack bean *urease* (4U) was mixed with 250 µl of different synthesized test compounds and standard of different concentrations [dissolved in DMSO/H_2_O mixture (1:1 v/v)]. The mixture was pre-incubated for 1 h at 37 °C in test tubes. 2 ml of 100 mM phosphate buffer (pH 6.8) containing 500 mM urea and 0.002% phenol red as an indicator were added in sample test tubes after pre incubation and again incubated at room temperature. Absorbance of reaction mixture was recorded by ELISA at 570 nm. Ammonium carbonate increased the pH of phosphate buffer from 6.8 to 7.7 which was produced from urea by *urease* enzyme and the end peak was measured by the colour of phenol red indicator [16].

**Table 5:** *Urease* inhibitory screening results of the synthesized compounds (**T**_**1**_–**T**_**20**_)

Compounds	% inhibition				IC_50_ (µg/ml)
	25 (µg/ml)	50 (µg/ml)	75 (µg/ml)	100 (µg/ml)	
**T**_**1**_	25.76	47.98	72.9	91.99	51.70
**T**_**2**_	40.45	49.54	55.67	67.57	53.04
**T**_**3**_	28.19	50.00	65.89	79.12	*54.01*
**T**_**4**_	35.45	49.45	63.67	79.45	50.52
**T**_**5**_	23.34	44.35	70.87	90.87	54.46
**T**_**6**_	38.45	50.34	65.61	80.89	47.02
**T**_**7**_	23.98	46.89	76.09	91.90	52.07
**T**_**8**_	22.45	39.58	68.58	92.67	56.43
**T**_**9**_	33.45	53.67	70.46	92.67	46.34
**T**_**10**_	27.90	46.89	70.90	89.80	51.90
**T**_**11**_	25.00	42.45	68.88	92.67	54.59
**T**_**12**_	43.56	46.57	52.56	61.56	58.06
**T**_**13**_	26.57	48.78	73.98	98.89	50.05
**T**_**14**_	41.70	46.23	50.49	57.78	67.02
**T**_**15**_	29.03	37.14	61.78	73.12	61.04
**T**_**16**_	45.76	49.10	53.87	59.12	52.85
**T**_**17**_	37.91	42.38	57.61	71.42	57.47
**T**_**18**_	40.90	46.79	55.89	67.9	54.30
**T**_**19**_	31.89	47.98	52.98	65.87	63.24
**T**_**20**_	28.98	49.09	69.09	81.90	52.34
**Thiourea**	29.98	46.76	67.78	76.78	*54.25*

Italics signifies the most active compound in comparison to the standard compound

The percentage inhibition of *urease* enzyme was calculated by using following formula:

$$ {\text{I}}\% = \frac{{{\text{A }}_{{{\text{control}}}} {-}{\text{A }}_{{{\text{sample}}}} }}{{{\text{A }}_{{{\text{control}}}} }} \times 100, $$

where A_control_ is the absorbance of the control; A_sample_ is the absorbance of the test compound.

#### Anticancer evaluation

**Fig. 12:**
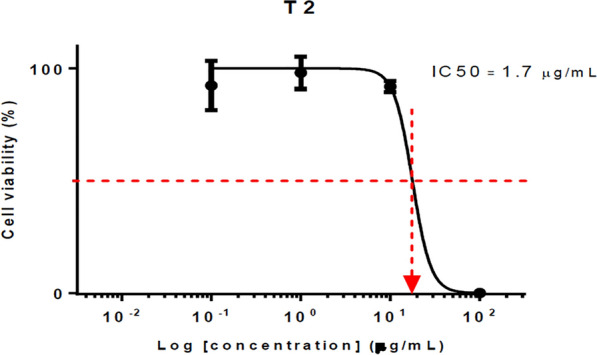
IC_50_ (µg/ml) of compound **T**_**2**_

**Fig. 13:**
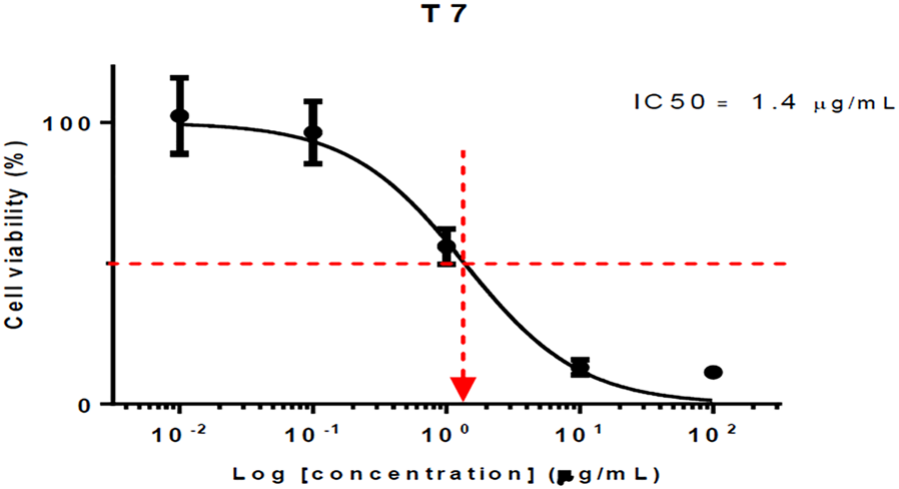
IC_50_ (µg/ml) of compound **T**_**7**_

**Fig. 14:**
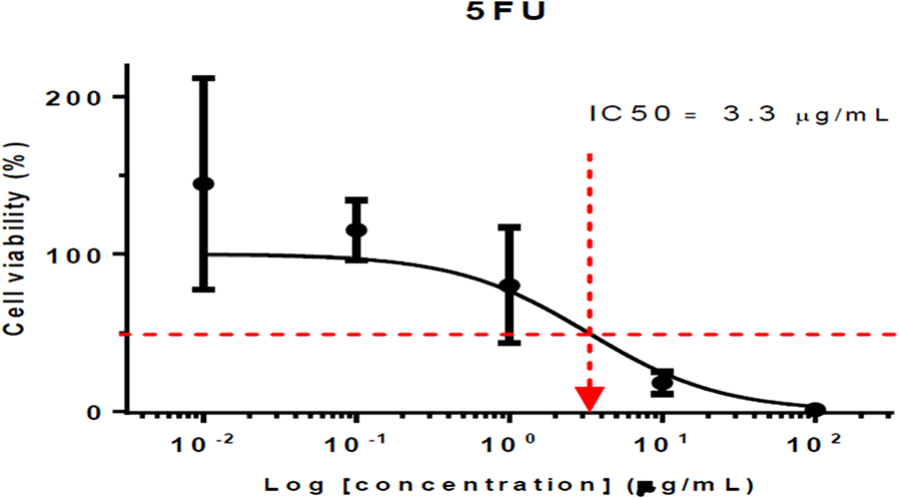
IC_50_ (µg/ml) of standard drug 5-FU

HCT116 (human colon cancer cells) were seeded at 2500 cells/well (96 well plate), allowed to attach overnight, exposed to the respective compounds for 72 h and subjected to SRB assay (570 nm). Data represent mean IC_50_ of at least triplicates. The compounds were all dissolved in DMSO as stock of 100 mg/ml. DMSO of < 1.5% did not result in cell kill. The highest concentration of each compound tested (100 μg/ml) contained only 0.1% DMSO. Compounds **T**_**2**_ (IC_50_ = 3.84 μM) and **T**_**7**_ (IC_50_ = 3.25 μM) exhibited the most potent anticancer activity against HCT116 cell lines as compared to standard 5-FU (IC_50_ = 25.36 μM) given in Table 6 and Figs. 12, 13, 14.

**Table 6:** Anticancer screening results of the synthesized compounds (**T**_**1**_–**T**_**20**_**)**

Compounds	IC_50_ (μM)	Compounds	IC_50_ (μM)
**T**_**1**_	> 241.16	**T**_**12**_	> 206.80
**T**_**2**_	*3.84*	**T**_**13**_	> 202.61
**T**_**3**_	16.56	**T**_**14**_	128.25
**T**_**4**_	7.79	**T**_**15**_	> 217.55
**T**_**5**_	> 198.12	**T**_**16**_	169.25
**T**_**6**_	112.00	**T**_**17**_	> 217.55
**T**_**7**_	*3.25*	**T**_**18**_	> 208.02
**T**_**8**_	> 232.20	**T**_**19**_	> 226.91
**T**_**9**_	> 218.47	**T**_**20**_	> 247.15
**T**_**10**_	173.90	**5-FU**	*25.36*
**T**_**11**_	> 222.66	**DMSO**	1.50%

Italics signifies the most active compound in comparison to the standard compound

## Conclusion

All the compounds were synthesized according to synthetic scheme under appropriate experimental conditions and analysed by elemental analysis, IR, mass, and ^1^H/^13^CNMR. The pharmacological potential was evaluated to study the effect of different substituents on antimicrobial, antioxidant and anti-*urease* activities. From the outcomes of the pharmacological studies it can be concluded that the substitution of tri-methoxy (**T**_**5**_) group increases the antibacterial activity whereas introduction of nitro (**T**_**17**_) group at *p-*position enhances the antifungal activity. The introduction of aldehyde (**T**_**2**_) and methyl (**T**_**3**_) at *p-*position of aromatic ring may increase the anti-*urease* as well as antioxidant activities. The substitution of *p*-aldehyde (**T**_**2**_) and *o*-hydroxy (**T**_**7**_) groups on the aromatic ring may enhance the anticancer activity against HCT116 cell line.

## Data Availability

The datasets used and/or analysed during the current study are available from the corresponding author on reasonable request.

## Abbreviations

IR: Infrared
NMR: Nuclear magnetic resonance
BS: *Bacillus subtilis*
PA: *Pseudomonas aeruginosa*
EC: *Escherichia coli*
CA: *Candida albicans*
AN: *Aspergillus niger*
DPPH: 2,2-Diphenyl-1-picryl-hydrazyl-hydrate
MCF-7: Michigan Cancer Foundation-7
HCT116: Human colon cancer cell line
5-FU: Fluorouracil
MIC: Minimum inhibitory concentration
Cipro: Ciprofloxacin
Amo: Amoxycillin
Flu: Fluconazole
IC_50_: Half maximal inhibitory concentration/median inhibitory concentration
SAR: Structure activity relationship
DNA: Deoxyribose nucleic acid
TLC: Thin layer chromatography
ATR: Attenuated total reflection
DMSO-d_6_: Di-methyl sulfoxide
ELISA: Enzyme-Linked Immuno Sorbent Assay
